# Carbohydrate Mouth Rinses before Exercise Improve Performance of Romanian Deadlift Exercise: A Randomized Crossover Study

**DOI:** 10.3390/nu16081248

**Published:** 2024-04-22

**Authors:** Tsung-Jen Yang, Yi-Jie Shiu, Che-Hsiu Chen, Sheng-Yan Yu, Ya-Ying Hsu, Chih-Hui Chiu

**Affiliations:** 1 Physical Education Center, Feng Chia University, Taichung City 407, Taiwan; andy32437@yahoo.com.tw; 2 Department of Physical Education and Sport Sciences, National Taiwan Normal University, Taipei 106, Taiwan; shiu880511@gmail.com; 3 Department of Sport Performance, National Taiwan University of Sport, Taichung 404, Taiwan; jakic1114@ntus.edu.tw; 4 Graduate Program in Department of Exercise Health Science, National Taiwan University of Sport, Taichung 404, Taiwan; j.yan0331@gmail.com; 5 Jaunan Elementary School, Miaoli 350, Taiwan; ndsl852@gmail.com

**Keywords:** ergogenic aids, eccentric exercise, peak power, strength training

## Abstract

(1) Background: This study compared the effects of mouth rinsing with a carbohydrate trial (CMR) and a placebo trial (PL) on concentric and eccentric contraction strength in multi-joint resistance exercise performance. (2) Methods: Twenty healthy adult men (age: 22.4 ± 3.7 years, body mass index: 26 ± 3.8, peak power: 378.3 ± 138.7 W) were recruited in this study. Participants were employed in a double-blind, randomized crossover design to divide participants into carbohydrate mouth rinsing trial (CMR) and placebo trial (PL). After warming up, participants used 6.6% maltodextrin (CMR) or mineral water (PL) to rinse their mouth for 20 s. Next, the participants underwent tests of maximum inertial Romanian deadlift resistance exercise comprising five sets of six reps, with 3 min rests between sets. After deducting the first repetition of each set, the mean values from the five sets were analyzed. (3) Results: The concentric peak power of the CMR trial was significantly higher than that of the PL trial (*p* = 0.001, Cohen’s d = 0.46), the eccentric peak power of the CMR trial was significantly higher than that of the PL trial (*p* = 0.008, Cohen’s d = 0.56), and the total work of the CMR trial was significantly higher than that of PL trial (*p* = 0.002, Cohen’s d = 0.51). (4) Conclusions: These findings demonstrate that mouth rinsing with carbohydrates before exercise can improve concentric and eccentric contraction strength in multi-joint resistance exercise performance.

## 1. Introduction

In the early 20th century, carbohydrates (CHO) were discovered to be a crucial source of energy for exercise [1]. During prolonged endurance exercise, carbohydrate intake helps maintain blood glucose concentrations, exercise reaction times, and technical performance [2,3]. However, even during shorter, higher-intensity exercise (e.g., <1 h, 75% VO_2max_), carbohydrate intake can improve exercise performance [1]. A study by Jeukendrup et al. found that exogenous carbohydrates could only provide 5–15 g as an energy source during the first hour of exercise. Such amounts have a very weak effect on delaying muscle glycogen depletion [4]. For this reason, muscle glycogen depletion is not considered a performance-limiting factor, and other factors may enhance performance during exercise.

Studies have had participants gargle an aqueous solution of CHO in the oral cavity for several seconds and spit it out. This method was associated with improved performance during endurance activities [5]. The first study to examine the effect of carbohydrate mouth rinsing on exercise performance was Carter’s research, which found that carbohydrate mouth rinsing significantly improved endurance exercise performance over a 60 min period [6]. During mouth rinsing, the CHO in the aqueous solution travels to the neural sensors in the oral cavity, which improves the performance of the nerve signals transmitted to the brain; this provides indirect evidence of the strengthening of the central nervous system effect [7]. Rinsing the mouth with a CHO solution and then spitting it out can also prevent gastrointestinal discomfort caused by the ingestion of CHO and the conflict between improving sports performance and the ingestion of CHO for those with weight control needs; this method also provides new reference for the supplementation of CHO in sports performance.

Although there is a lot of evidence to suggest that carbohydrate mouth rinsing can be effective in enhancing endurance exercise performance, studies on the effects of CHO-solution mouthwashes on short-term, high-intensity exercise and resistance training have yielded inconsistent results. Krings et al. found that CMR increased peak power during a 15 s bicycle sprint [8]. For resistance exercise, Decimoni et al. (2018) discovered that rinsing the mouth with a CHO solution before resistance exercise can reduce fatigue and enable weightlifters to lift heavier loads during resistance exercise [9]. Another study suggested that carbohydrate mouth rinsing was effective in increasing the number of repetitions of leg press and bench press [10]. In contrast, other studies have indicated that, after mouth rinsing with a CHO solution, the participants’ maximum power, average power, and fatigue did not differ significantly for 30 s maximum-intensity sprints [11]. For resistance exercise, mouth rinsing with a CHO solution also did not affect bench press one-rep max, upper-limb muscle endurance, or other anaerobic metabolic capacities [12]. Another study observed that gargling different doses of CHO solutions (6%, 12%, and 18%) before resistance exercise did not improve maximal muscle strength or muscle endurance [13]. These previous studies mainly focused on maximal strength, number of repetitions, and muscle fatigue during exercise. Up to the present, no study has examined the effect of carbohydrate mouth rinsing on eccentric performance. Since the eccentric training increases more muscular strength and power [14], increasing peak power during eccentric training seems to further enhance the effects of eccentric training on muscle strength.

Flywheel resistance exercise, a type of eccentric load training, has been widely discussed and used for various sports. In flywheel resistance exercise, inertial power generated by the flywheel during muscle acceleration and deceleration provides resistance in the eccentric and concentric phases, respectively [15]. The flywheel has been demonstrated to improve health and athletic performance and prevent sports injury [16]. For these reasons, the instrumentation used in this study allows us to understand both concentric and eccentric muscle contraction ability during exercise. As these abilities improved, the quality of training can be enhanced more effectively.

For lower extremity multi-joint exercises, the Romanian deadlift (RDL) has been found to improve strength in the hamstrings muscle groups as well as increase the sprinting speed [17]. Therefore, in this study, the RDL was used for multi-joint exercise. No studies have examined the effect of rinsing the mouth CHO solutions on the performance of concentric and eccentric contraction strength in multi-joint resistance exercise. For this reason, this study investigated the effect of carbohydrate mouth rinsing (CMR) on RLD resistance exercise performance. The hypothesis of this study is that CMR can effectively enhance concentric and eccentric contraction strength during RDL training.

## 2. Materials and Methods

### 2.1. Study Design

**Figure 1:**
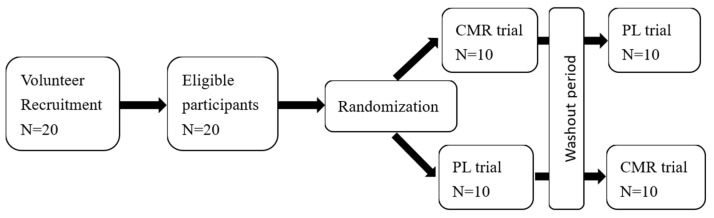
CONSORT diagram and study design.

This study conducted a randomized, crossover trial with a double-blind experimental design. All participants were subjected to two experimental conditions, one with CMR and the other with a placebo trial (PL). The CONSORT diagram and study design is shown in Figure 1. The CMR was a colorless and odorless 6.4% maltodextrin solution, and mineral water was used for the PL. All mouthwashes were first prepared in the laboratory, numbered, and then taken to the experiment field to ensure that the staff in the field test did not know which trial was assigned. After at least two introductory sessions, the participants were randomly divided into two experimental sequences, CMR-PL or PL-CMR, by using a crossover design. The second phase of the experiment was held after 7 days of rest and recovery to eliminate the effects of muscle fatigue and delayed muscle soreness.

### 2.2. Participants

This study recruited 20 healthy adult men (age: 22.4 ± 3.7 years, height: 171.3 ± 5.9 cm, weight: 76.4 ± 11 ± kg). The inclusion criteria were (1) regular weight training habits, (2) familiarity with Romanian deadlift movements, and (3) no previously known hypertension, diabetes, kidney or heart disease, or sports injuries such as lacerated knee ligaments, inflammation and rupture of tendons, strained biceps femoris or quadriceps, and strained biceps within the 6 prior months. Those who (1) did not have regular exercise habits, (2) were unfamiliar with Romanian deadlift movements, (3) had certain health problems that prevented them from performing exercise, and had an injury from which they had not recovered for more than 6 months were excluded. Before the experiment, the researchers explained the experimental procedure to the participants and obtained their informed consent. This study was approved by the Human Subject Research Ethics Committee of Jen-Ai Medical Foundation (111-09) and registered in ClinicalTrials.gov (Date: 6 August 2023; ID “NCT05900349”; https://register.clinicaltrials.gov, accessed on 24 March 2023). This study was conducted in accordance with the Declaration of Helsinki.

### 2.3. Study Setting

The experiments were conducted at the Sports Science Research Center of the National Taiwan University of Sports. We controlled the temperature and humidity (26 °C and 60% RH) of the Sports Science Research Center via the air conditioner. This study commenced on March 2022, and supplementation and sport performance tests were completed in October 2022. All participants completed the experiment, and no participant was excluded from the experiment.

### 2.4. Protocol

#### Pre-Test

Two pre-tests were conducted before the formal experiment. The pre-tests involved familiarization with the experimental apparatus, practicing the movements, and testing with different inertia loads. The load resistance was 0.010, 0.025, 0.050, or 0.075 kg, and the participants’ peak power was used as the experimental load resistance. The pre-tests were separated by 7 days, and the first phase of the formal experiment and the last pre-test were also separated by 7 days to allow for adequate recovery of muscle performance.

The participants used an Exxentric inertial resistance training machine (Exxentric kbox 4 Pro, Stockholm, Sweden) to complete the Romanian deadlift movement test. With the maximum inertial resistance measured during the pre-test being used as the resistance load during the experiment, the participants underwent a maximum inertial resistance exercise comprising five sets of five reps, with a 3 min rest between.

The second pre-test involved the same procedure as the first pre-test but with the participants using the load resulting in the highest power output identified during the first pre-test; the pre-tests were conducted at least 7 days apart. Before each test, the participants underwent a standardized warm-up supervised by the individual conducting the experiment, which included riding on a stationary bicycle with a speedometer for 5 min (at a manageable intensity, which was in the 10–12 range on the perceived exertion (RPE) scale, with the full range being 6–20), a joint range-of-motion warm-up, and dynamic stretching for 3 min. Before the warm-up, the scale was explained. We measured participants’ RPE using the Borg RPE scale, ranging from 6 (no exertion) to 20 (maximum exertion). 

### 2.5. Experimental Procedure

The participants were required to arrive at the laboratory at 3:30 p.m. on the day of the experiment, fast for at least 3 h beforehand, and avoid high-intensity training (e.g., weight training, endurance training, and high-intensity interval training) for 3 days before the experiment. The contents of the diet and the time of consumption 24 h before the first experiment were recorded using photographs. The staff reminded the participants to eat the same food at the same time during the second formal experiment, especially for breakfast and lunch on the day of the experiment. The nutritional composition of breakfast and lunch was 13.9 ± 1.4% protein, 50.3 ± 12.7% carbohydrate, 35.8 ± 5.2% lipid, and 1423.6 ± 111.4 kcal. These nutrient calculations were performed by an experienced dietitian. Also, all participants were asked to avoid caffeine, alcohol, energy drinks, and any form of stimulant or other substance that would affect exercise performance. 

Before the experiment, the participants performed a standardized warm-up, comprised of riding on a stationary bicycle for 5 min, joint range-of-motion warm-up and dynamic stretching for 3 min, and one set of eight reps of Romanian deadlifts with the flywheel; the load resistance was 0.01 kg. The participants rinsed their mouths with either 25 mL of CHO solution or the placebo solution for 20 s and spit the solution into a designated container after rinsing. Next, the participants used the inertial resistance exercise machine to complete the Romanian deadlift movement test. With the maximum inertial resistance measured during the pre-test being used as the resistance load during the experiment, the participants completed a maximum inertial resistance exercise comprised of five sets of five reps, with a 3 min rest in between. A 0.6 kg training bar was attached to the inertial resistance exercise machine during each testing session. 

To standardize the participants’ range of motion during testing, a piece of tape was placed at the midpoint between the tibial trochanter and talus to indicate the range of motion for the Romanian deadlifts, with the endpoint being full hip extension. The process was as follows:

1. Participants stood over the tether with their feet hip-width apart, toes pointing forward, and head and eyes facing straight ahead.
2. Hands were positioned shoulder-width apart, naturally lowered, with a positive grip on the training bar.
3. The training bar was connected to the tether, which was connected to the flywheel shaft through a hole in the platform, and the movement began in the bent position (i.e., at the designated starting point of the range of motion). The participants pushed back on their gluteus maximus and kept their back straight, their shoulders pulled back, and their chest up.
4. Each movement started with the concentric phase and ended with the eccentric phase.
5. In the concentric movement phase, the crossbar was pulled along the thigh, and the tether was pulled to put the flywheel in motion. Participants contracted the gluteus maximus, hamstrings, and lower back muscles until the hip joint was fully extended to the end position.
6. During the eccentric movement stage, the flywheel rotated, and the motion decelerated from the end position through centrifugal muscle contraction. Participants then completed the motion and returned to the starting position through a braking motion.
7. These steps were repeated until the test was complete.
8. The participants were instructed to not shrug their shoulders when their hip joints were fully extended and not allowed to flex their ankles.
9. The participants began resisting the inertia in the first third of the concentric movement and then stopped moving at the end of the range of motion with maximum effort; the participants were instructed to perform the concentric movements quickly.

Before each set, participants performed one test movement to increase the power of the flywheel, followed by five test movements. During the test, the participants were verbally encouraged to complete the test to the best of his ability. These initiated the movement of the flywheel and increased its speed, and the resulting data were excluded from statistical analysis. In the second experiment, which was conducted 7 days later, the participants repeated the steps from the first experiment. 

The participants’ test data were measured using a Kmeter, fed back to the system, and transferred to an Android device (ASUS ZenFone 6, ASUS, Taipei, Taiwan) over Bluetooth for storage. The data were then exported to a computer in comma-separated value format for statistical analysis. The researchers analyzed data on the following seven factors measured using the Kmeter feedback system: power, concentric peak power, eccentric peak power, average force, average speed, peak speed, and total work. Similar research methods have been published in the previous study [18].

### 2.6. Carbohydrate Solution and Placebo

In the experiment, the carbohydrate solution was prepared by dissolving 6.4 g of colorless and odorless maltodextrin in 93.6 mL of water. The mixtures were then adjusted to a 6.4% maltodextrin solution for the participants to rinse their mouths. The placebo group rinsed their mouths with drinking water. The mouthwash used in this study was similar to those used in a previous study, which found that CMR was effective in increasing the fatigue index in taekwondo athletes [19]. All carbohydrate solutions were prepared by the principal investigator of the research project before the experiment, and they were similar to the placebo in appearance and taste to ensure that the participants could not distinguish them. Mouth rinsing was defined as distributing the carbohydrate solution around the mouth for 20 s [20], followed by spitting it out into a designated container. The most commonly used carbohydrate solutions are glucose and water-soluble maltose dextrin at a 6.4% concentration [21], which are used for 15–20 s before exercise. Thirteen participants correctly guessed they were on carbohydrate (65%) and nine participants correctly guessed they were on placebo (45%).

### 2.7. Lower Limb Power Test

Following a 10 min rest period, individuals engaged in a session comprised of 6 repetitions and 5 sets of Romanian deadlift (RDL) exercises, utilizing the non-gravity-dependent flywheel inertial device (k-Box 4, Exxentric^®^, Stockholm, Sweden). Each set was separated by a 3 min rest period. The Exxentric kMeter, linked to its application via Bluetooth, was employed in every RDL assessment to document average power (W), average force (N), peak concentric power (W), peak eccentric power (W), and total work (KJ). This apparatus, previously validated in various performance evaluations, showcased reliable metrics [22]. Participants wore wrist-based heart rate monitors for real-time tracking of heart rate in beats per minute (bpm). These non-intrusive devices provided precise cardiovascular data throughout the physical activity. Concurrently, participants self-rated their exertion using the rating of perceived exertion (RPE) scale, ranging from 6 (no exertion) to 20 (maximum exertion). This methodology facilitated the correlation of objective heart rate measurements with subjective exertion ratings, offering a comprehensive evaluation of the participants’ physical demands. In the CMR trial, 12 out of 20 (60%) participants inaccurately identified they were rinsing with carbohydrate or placebo. Conversely, in the PL trial, 11 out of 20 (55%) participants made incorrect guesses regarding the carbohydrate or placebo.

### 2.8. Sample Size Calculation

The G*Power 3.1.9.6 [23] software was used to determine the required sample size based on data from a previous study [10]. This study suggested that CMR increases the number of repetitions during bench press exercise in recreational resistance-trained males (effect size = 0.78). Upon analyzing the aforementioned data using a paired *t*-test, it was determined that a total sample size of 8 would be required to achieve 80% power in detecting significant variances in the total number of repetitions during the bench press exercise. The alpha level was set at 0.05, and a correlation coefficient of 0.5 was assumed. This sample size calculation was designed to detect an effect size of 0.78 (Cohen’s d) using a paired *t*-test to compare the two trial conditions. Based on these results, the recruitment of 20 participants in this study should be sufficient to explain the results of the data.

### 2.9. Statistical Analysis

The data are presented as means ± standard deviations, and the analysis was performed using SPSS (version 20, Chicago, IL, USA). The Shapiro–Wilk test was conducted to test the normality of the data. A paired-samples *t*-test was used to compare the average power, concentric peak power, eccentric peak power, average force, average speed, peak speed, and total work in the five Romanian deadlift sets between the CMR and PL groups. The before-and-after differences in heart rate and rating of perceived exertion (RPE) were analyzed using a two-way ANOVA with repeated measures. The study assessed the impact by calculating the effect size through Cohen’s d formula [24] to measure the observed effects’ magnitude. The significance was *p* < 0.05.

## 3. Results

### 3.1. Flywheel Exercise Loading

The average isoinertial load was 0.061 ± 0.02 kg·m^−2^. Eleven of the twenty participants had an isoinertial load of 0.05 kg·m^−2^, six had a load of 0.075 kg·m^−2^, and three had a load of 0.1 kg·m^−2^. The peak power in the pre-test was 378.3 ± 138.7 W.

### 3.2. Concentric and Eccentric Peak Power

**Figure 2:**
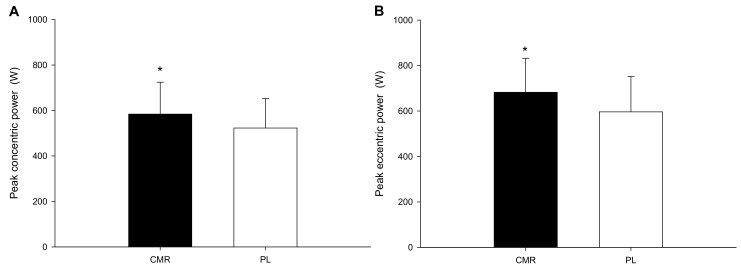
Peak concentric and eccentric power. The peak concentric power (**A**) and peak eccentric power (**B**) of the CMR and PL trials were compared. * CMR was significantly higher than those for the PL.

Comparing the peak concentric power (Figure 2A) between the two trials, the results showed that the CMR trial had significantly higher peak concentric power compared to the PL trial (CMR: 584.1 ± 136.0 W; PL: 522.9 ± 122.9 W; *p* = 0.001, Cohen’s d = 0.46). The peak eccentric power (Figure 2B) was significantly higher in the CMR group than in the PL group (CMR: 682.4 ± 145.4 W; PL: 596.4 ± 151.1 W; *p* = 0.008, Cohen’s d = 0.56).

### 3.3. Average Power, Average Force, and Total Work

**Figure 3:**
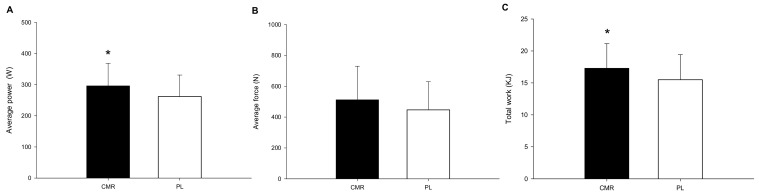
The average power (**A**), average force (**B**), and total work (**C**) of the CMR and PL trials were compared. * CMR was significantly higher than those for the PL.

Figure 3A showed the comparison of the average power between the CMR trial and the PL trial. The average power of the CMR trial was significantly higher than that of the PL trial (CMR: 296.2 ± 70.1 W; PL: 261.7 ± 67.1 W; *p* = 0.001, Cohen’s d = 0.49). The two trials did not differ significantly in terms of average force (CMR: 512.3 ± 211.0 N; PL: 446.7 ± 177.5 N; *p* = 0.186, Cohen’s d = 0.32) (Figure 3B). Total work (Figure 3C) in the CMR trial was significantly higher than that in the PL trial (CMR: 17.3 ± 3.7 KJ; PL: 15.5 ± 3.8 KJ; *p* = 0.002, Cohen’s d = 0.51).

### 3.4. Heart Rate and RPE

**Figure 4:**
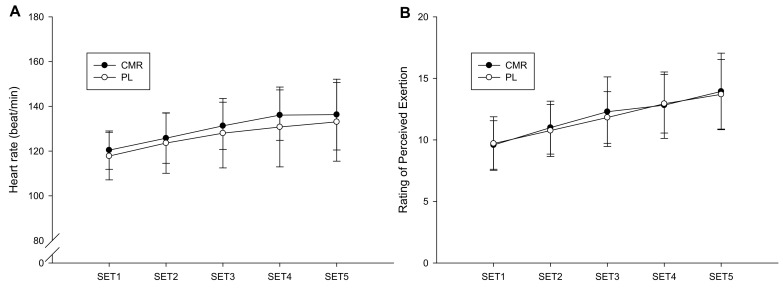
Comparison of heart rate (**A**) and rating of perceived exertion (**B**) between the CMR and PL trials.

Figure 4A presents the heart rates of the CMR and PL trials. The trials did not differ significantly in their heart rate over the five sets (trial × time = 0.809, trial = 0.245, time < 0.001). The trials also did not differ significantly in terms of average heart rate over the five sets (CMR: 130.0 ± 9.6 bpm; PL: 126.6 ± 14.1 bpm; *p* = 0.245). The RPE values of the groups are presented in Figure 4B. The groups did not differ significantly in terms of RPE over the five sets (trial × time = 0.492, trial = 0.683, time < 0.001). The average RPE of the trials over the five sets was 11.9 ± 2.4 for the CMR trial and 11.8 ± 2.0 for the PL group (*p* = 0.683).

## 4. Discussion

The results indicate that mouth rinsing with the carbohydrate solution before RDL resistance exercise significantly improved in exercise performance in terms of eccentric peak power, concentric peak power, average power, and total work with a moderate effect size compared with the placebo treatment. These results may indicate that mouth rinsing with carbohydrates before exercise can improve resistance exercise performance.

With regard to whether CMR before exercise can improve performance in explosive power sports, the results are inconsistent. Krings et al. had participants rinse their mouths with a carbohydrate solution before a full-power bicycle dynamometer sprint (15 s) and discovered that mouth rinsing increased peak power [8]. However, other studies have indicated that after mouth rinsing with a CHO solution and a 30 s maximum-intensity bicycle ergometer sprint, the participants’ maximum power, average power, and fatigue did not differ significantly [11]. Mouth rinsing with a CHO solution also did not affect bench press one-rep max, upper-limb muscle endurance, or other anaerobic metabolic capacities [12]. For this reason, some scholars have noted that mouth rinsing with a CHO solution does not contribute to the energy supply of the metabolic system required during high-intensity exercise [21]. Unlike other studies, this study explored exercise capacity during inertial resistance exercise. CMR increased concentric peak force, eccentric peak force, and total work during inertial resistance exercise. From these data, it appears that mouth rinsing with carbohydrates prior to performing resistance exercise is a viable way to enhance strength during resistance exercise.

Carbohydrates may improve performance during inertial resistance exercise through three mechanisms. First, chemoreceptors from the tongue and oral cavity are stimulated by the CHO solution in the oral cavity, carry a message to the nucleus of the solitary tract, and act on the ventral posterior thalamus of taste, which transmits neurons in the insula cortex and excites the motor cortex. Second, CHO in the human oral cavity produces central neural responses, including the activation of the insula, frontalis, orbitofrontal cortex, and striatum, which comprise the reward and motor control areas of the brain, and thus improves motor performance [21]. Third, Krings et al. (2017) observed that gargling with a CHO solution before short-term, high-intensity bicycle sprints significantly decreased the fatigue index [8]. Studies have also demonstrated that mouth rinsing with a CHO solution can increase pleasure through the gray matter of the spinal cord lamina I, thereby mitigating the effects of RPE, improving motor neurofeedback related to emotion and motivation, and increasing power during exercise [7]. Although this study did not observe a significant difference in RPE between the groups, the results of other studies suggest that the cause of performance improvement in this study may be the increase in cell production and the stimulation of the nerve receptors in the oral cavity after mouth rinsing with a CHO solution.

The main advantage of the flywheel inertial resistance exercise is related to the high mechanical load during the eccentric phase. Greater eccentric loads may cause motor units to recruit muscle fibers and, thus, result in a postactivation potential reaction during certain forms of exercise [25]; this can improve performance during stretch-shortening cycles and explosive exercises (such as vertical jumps, horizontal jumps, and sprinting). The results of this study indicate that mouth rinsing with a CHO solution can improve RDL exercise performance after exercising with an inertial resistance device. On the other hand, there are some past studies suggesting that the CMR methods have no effect on performance in resistance exercise [11,12,13]. During eccentric contraction, the control of the nervous system and the activation of the cerebral cortex are different from concentric contraction [26]. These different physiological as well as cerebral responses may have contributed to the present study’s finding that CMR is effective in enhancing resistance exercise performance. This study provides objective, scientific evidence demonstrating that mouth rinsing with a CHO solution before inertial or resistance exercise improves training quality. This finding can provide athletes or coaches of explosive power sports with reference to improve training interventions and preparation regimens for competitions. For researchers, it is possible to further investigate whether the use of the CMR method affects the physiological response to eccentric exercise.

The strength of this study, and what makes it different from other past studies, is that CMR was also found to have a significant improvement on peak power during eccentric training. Although the researchers attempted to ensure rigor, this study has several limitations. First, this study did not use electroencephalography or functional magnetic resonance imaging to explore brain activity after CMR, preventing observation of the exact mechanism through which inertial resistance exercise improved after CMR. However, this study still provides solid scientific evidence that CMR prior to inertial resistance exercise significantly improves exercise performance at the RDL, with a moderate effect size. Second, even though we tried to make the colors and flavors as similar as possible between the two trials, half of the participants were able to guess the contents of the supplements. Such a result may represent an unsatisfactory double-blind experiment. However, during the experiment, the participants were encouraged to perform the movements to the best of their ability. In fact, some of the past studies used sweeteners as a placebo [8,9,12], and some of the studies used mineral water as a placebo [11,13,19]. However, none of these studies investigated whether participants could be effectively distinguished into a carbohydrate trial or a placebo trial. We were also unable to distinguish whether the participants in the past studies were able to successfully distinguish the experimental trial from the control trial. On the other hand, all research data were measured using the Exxentric kMeter, and the values were not immediately available to the participants. This study guaranteed the accuracy of outcome measures by employing device-based measurements to precisely reflect the participants’ performance. During the test, the participants were verbally encouraged to complete the test to the best of his ability. Verbal encouragement ensures that all participants are performing to the best of their ability at the time of the test. Finally, we didn’t measure the weight of the actual gargle solution that was spit out. A small amount of the gargle solution may be swallowed by the participant, but this should not affect the effectiveness of the gargle on exercise performance. 

## 5. Conclusions

The main finding of this study is that mouth rinsing with a carbohydrate solution before RDL resistance exercise significantly improved eccentric peak power, average force, peak speed, and total work. These results suggest that simple mouth rinsing with carbohydrates before short-term, high-intensity exercise improves exercise performance. Subsequent studies can explore female participants and use other training equipment to obtain experimental results on the effects of CMR on efficacy in moderate- and short-term, high-intensity exercise.

## Data Availability

All relevant materials are presented in the present manuscript.
